# Innate Immune Response in Implant-Associated Infections: Neutrophils against Biofilms

**DOI:** 10.3390/ma9050387

**Published:** 2016-05-18

**Authors:** Ulrike Dapunt, Gertrud Maria Hänsch, Carla Renata Arciola

**Affiliations:** 1 Center for Orthopaedics, Trauma Surgery and Spinal Cord Injury, Heidelberg University Hospital, Schlierbacher Landstrasse 200a, Heidelberg 69118, Germany; 2 Institute for Immunology, Heidelberg University, Im Neuenheimer Feld 305, Heidelberg 69120, Germany; maria.haensch@urz.uni-heidelberg.de; 3 Research Unit on Implant Infections, Rizzoli Orthopaedic Institute, Bologna 40136, Italy; carlarenata.arciola@ior.it; 4 Department of Experimental, Diagnostic and Specialty Medicine, University of Bologna, Bologna 40126, Italy

**Keywords:** implant infections, biofilm, innate immune response, neutrophils, GroEL, Staphylococcus, orthopedics

## Abstract

Biofilm has been recognized as a well-protected form of living for bacteria, contributing to bacterial pathogenicity, particularly for opportunistic species. Biofilm-associated infections are marked by their persistence. Extensive research has been devoted to the formation and composition of biofilms. The immune response against biofilms remains rather unexplored, but there is the notion that bacteria within a biofilm are protected from host defences. Here we glance at the mechanisms by which neutrophils recognize and face biofilms in implant infections and discuss the implications of this interplay, as well as speculate on its significance.

## 1. Introduction

Even though major improvements have been made over the last years, bacterial infections of implants are still a feared complication in the field of orthopaedics [1,2]. These infections are particularly difficult to diagnose and frequently require prolonged treatments [3,4], because bacteria are capable of forming sessile communities on the implant surface and embed themselves in a slimy matrix (extracellular polymeric substance), the so-called “biofilm” [5].

Owing to the outstanding work by Bill Costerton and other renowned scientists, we now know that biofilm formation is a trendy lifestyle for bacteria and a well-functioning tool by which opportunistic bacteria acquire pathogenic potential [6,7,8,9,10,11]. Biofilm formation has also been described in other environments (first, for water-dwelling bacteria adhering on the rocks) and usually helps surviving in disadvantageous conditions, such as lack of nutrition [12,13,14,15]. In the human body, however, biofilm formation is thought to play a different role, namely protection against the host’s defence mechanisms. Some biofilms are well tolerated by the immune system (such as mucosal biofilms, for example) [16], whereas in other settings, like in the case of artificial joint replacement in orthopaedics, bacterial biofilms are thought to be the main reason for persistent and highly destructive infections [17,18,19,20], whose etiologic agents are prevalently Gram-positive species belonging to the *Staphylococcus* genus, among which particularly *Staphylococcus aureus* and *Staphylococcus epidermidis* [19,20,21].

Furthermore, these infections lead to a loss of bone substance, which results in loosening of the implant and hence the need to perform implant replacement surgery. This procedure often leads to debilitating results, and elicits psychological stress in affected patients, as well as high socioeconomic costs due to the protracted medical and surgical treatments [20,21,22,23,24]. Due to these detrimental effects, and because an ever increasing number of prostheses is implanted every year, research in this particular field is considered highly important. Furthermore, since most patients with implant infections unfortunately require surgery, the infection site becomes accessible and tissue samples can be obtained to study the inflammatory process.

## 2. Systemic and Local Immune Response in Biofilm Infections

An association between bacterial infection and bone loss has been well established. However pathomechanisms leading to bone degradation are still unclear [22,23]. Therefore, the aim of our research group during the last couple of years was to evaluate the process linking bone infections with bone loss.

In this context, we studied the systemic and local immune responses in patients suffering from implant infections. Standard systemic markers of an infection, such as elevated C-reactive protein concentration and/or white blood cell count, are known to be frequently unreliable in the case of implant-infections [25,26]. However, we evaluated an activation of T-cells (downregulation of CD28 and upregulation of CD11) in peripheral blood and found that activated T-cells were only in patients with implant infections [27], a rather surprising finding since T-cells are not thought to be chiefly involved in bacterial infections. Accordingly, when evaluating tissue samples from the infected site, we found infiltration of T-cells, as well as enhanced gene expression of CD3 [27].

In the local wound lavage, T cells were activated, particularly CD8+ cells [28]. Moreover, T cells recovered from the infected site were terminally differentiated and produced interferon gamma, a cytokine known to enhance functions of phagocyte cells, drawing the conclusion that infiltrated T cells support the local immune defence [29]. Aside from the T-cell response, we mainly looked at polymorphonuclear leukocytes (PMN, neutrophils), since these cells are considered to be the first-line defence against bacterial infections. We found a massive infiltration of activated neutrophils at the infected site, indicated by an upregulation of adhesion proteins (CD11b, CD18), Fc-receptors, MHC class II molecules, or the chemokine receptors CXCR6 [30,31]. Additionally, neutrophils showed an altered functional response, namely an enhanced production of oxygen radicals and a reduced chemotactic activity [32].

**Figure 1:**
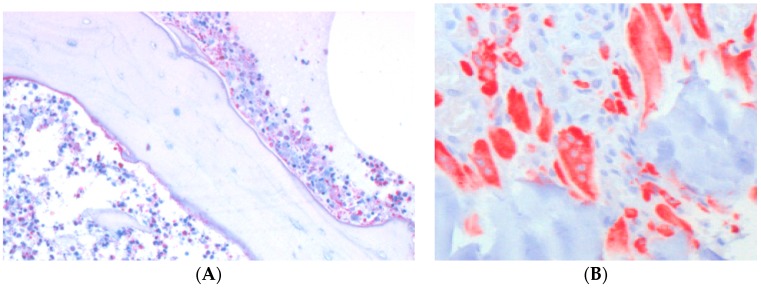
A dense infiltrate of neutrophils (**A**) can be detected in bone infections, as well as osteoclasts (**B**), red, after staining for cathepsin K.

Importantly, the number of neutrophils at the infected site correlated with the number of bone-resorbing osteoclasts (Figure 1A,B), which supports the idea that a persistent pro-inflammatory response is generated by immune cells and is doomed to end up with osteoclast generation and bone degradation [33].

Following this notion further, we evaluated expression of inflammatory cytokines in tissue samples and found that certain cytokines—chiefly CXCL8 (IL-8), CCL3 and CXCL2 (the macrophage inflammatory proteins MIP1α and MIP2α), and S100A9 (MRP14)—were elevated in patients suffering from implant infections [34,35,36]. Since MRP14 is an abundant content in neutrophils, we also investigated MRP14 plasma concentrations and found these to be elevated, advocating MRP14 as a promising future diagnostic marker in peripheral blood [35].

In summary, we are inclined to believe that, contrary to the perception that bacteria embedded in biofilms are not recognized by, and sheltered from, the immune system [37,38,39], polymorphonuclear leukocytes respond to bacteria in biofilms. Interestingly, it has been shown that most implants are colonized by bacteria without causing symptoms of an infection [40]. Since biofilms represent the preferred lifestyle of bacteria, it is more than likely that neutrophils are faced with developing biofilms. Perhaps biofilm infections are more common than previously presumed and are usually effectively cleared away without us noticing.

## 3. How Neutrophils Recognize Biofilms

Starting from the consideration that the immune system recognizes and responds to biofilms, the next question of interest is “how” biofilms are recognized.

As previously pointed out, biofilm formation is the result of a genetically driven process that is initiated by bacteria attaching to a surface. Once bacteria switch to their biofilm form, they exhibit an altered behavior; for example, acquiring increased resistance towards antibiotics and biocides, and producing an extracellular polymeric substance (EPS) [41]. There are several theories why an increased tolerance to antibiotics might occur, such as conventional resistance mechanisms (β-lactamase), but also upregulated efflux pumps, mutations in antibiotic target molecules, and the possibility of a more dormant group of bacteria within the biofilm are thought to contribute [42]. Main constituents of the EPS are the polysaccharide intercellular adhesin (PIA), extracellular-DNA, proteins, and amyloid fibrils [9]. PIA is a poly-β(1-6)-*N*-acetylglucosamine (PNAG), partially deacetylated, and positively charged, whose synthesis is controlled by *icaADBC* locus (reviewed in [43]). Not only the expression of *icaADBC* is affected by the *milieu* conditions or subjected to switch on/off mechanisms [44,45,46], but also there are *ica*-independent mechanisms of biofilm production that have been detected and described (reviewed in [47,48]). Indeed, a number of proteins localized in the extracellular matrix of biofilms have been identified that can generate PIA-independent biofilms (reviewed in [48]).The entire process is regulated via the so-called quorum-sensing (QS) molecules (also termed “autoinducers”), which represent a method of bacterial communication [49,50,51]. Interfering with the QS system is a much debated strategy to combat biofilm-related infections [48,49,50]. QS molecules and the composition of the EPS are known to be different among bacteria species. The EPS has numerous functions: it serves as a scaffold for the three-dimensional structure of the biofilm, protects the bacteria against environmental stress, facilitates horizontal gene transfers, and sequesters nutrients from the surroundings [52,53,54,55].

Generally, invading bacteria are faced with the complex host defence system of different cells, numerous cellular receptors, signalling pathways, and effectors molecules. This abundance of defence mechanisms is required since bacteria species also differ with regard to their susceptibility to the host defence [56,57,58,59,60]. We will focus on polymorphonuclear leukocytes, since these cells can be found in abundance at the infected site, represent the first line of defence against infections and are, therefore, crucial for the host defence against bacteria.

When neutrophils sense a localized infection, they emigrate from blood vessels and migrate actively through the tissue to the site of infection in a well-controlled fashion [59,60,61]. Once arrived, neutrophils phagocytose bacteria and have a repertoire of cytotoxic substances at their demand to kill bacteria intracellularly or extracellularly, among their reactive oxygen species (ROS) [62,63].

Importantly, neutrophils do not recognize bacteria in an antigen-specific manner. Rather, they are equipped with numerous receptors to recognize evolutionary-conserved surface molecules, the so-called pathogen-associated molecular patterns (PAMPs) and microbe-associated molecular patterns (MAMPs) [64,65,66].

With regard to free-swimming/“planktonic” bacteria, mechanisms leading to phagocytosis and killing have been thoroughly investigated. Phagocytosis of planktonic bacteria requires a process termed “opsonization”, whereby bacteria are coated with an antibody and complement (complement C3b/C3bi). Receptors for immunoglobulin G (CD16, CD32 and, following activation, CD64, as well) mediate phagocytosis and intracellular killing together with the complement receptors (CR1, CR3) [56,61,67].

**Figure 2:**
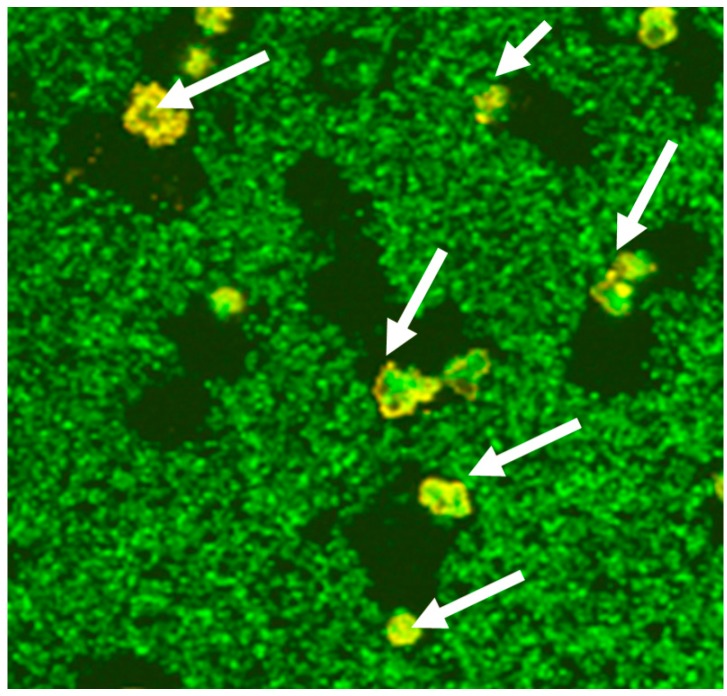
*Staphylococcus epidermidis* biofilms were grown (green). Neutrophils (arrows) attacked and phagocytosed the biofilm, as seen by the spaces in between the biofilm.

Interestingly, opsonization does not seem to be required for recognition of biofilms (Figure 2) [68] and we, therefore, hypothesized that the slimy substance, the EPS, might contain activating factors; a notion which is also supported by data by others [69,70].

We demonstrated that neutrophils were in fact activated by EPS extracted from *Staphylococcus epidermidis* biofilms, which was exhibited as release of lactoferrin and upregulation of the activation-associated adherence proteins CD11b/CD18 and of CD66 [71]. Furthermore, we recently investigated potential activating molecules within the EPS in further depth. First, obvious candidates, such as LTA (lipoteichoic acid), PIA (poly-*N*-acetyl(1-6)β-glucosamine), or LPS as a contaminant, were removed from the EPS. Digestion of the EPS by trypsin resulted in a loss of activation, thus directing our search towards a protein.

**Figure 3:**
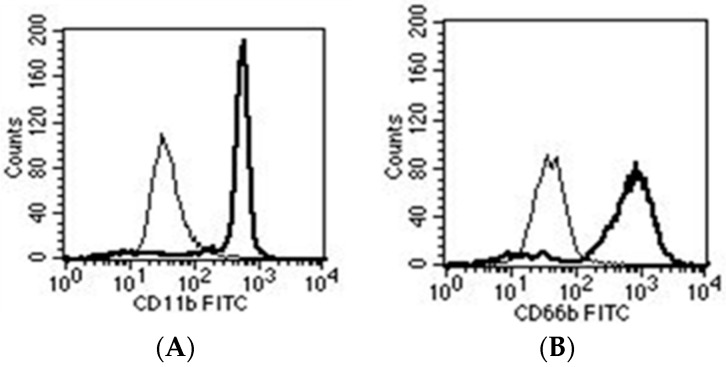
After stimulation with GroEL, neutrophils upregulated the activation markers CD11b (**A**) and CD66b (**B**) as shown by cytofluorometry (thin line: control, thick line: after stimulation with GroEL).

We identified the protein GroEL (a bacterial heat shock protein) as an activating factor, since stimulation of neutrophils with recombinant GroEL resulted in an increased oxygen radical production and in an upregulation of the cell surface activation markers CD11b and CD66b (Figure 3A,B) [72]. GroEL is a highly-conserved protein, shares homologies with the human heat shock protein 60 and is essential for protein folding [73]. Of note, it has been shown in the literature that bacteria cannot survive without GroEL [74], indicating this protein as a favorable and strategic target point for neutrophils against various bacteria species. Therefore, GroEL can be regarded as a “pathogen-associated molecular pattern” for the host response. Accordingly, we identified the pattern-recognition receptor TLR4 as a possible receptor for GroEL.

## 4. Neutrophil Extracellular Traps (NETs)

Another method for neutrophils to fight bacteria is the release of DNA-based traps, also called “netosis” or “NET (neutrophil extracellular traps) formation”.

**Figure 4:**
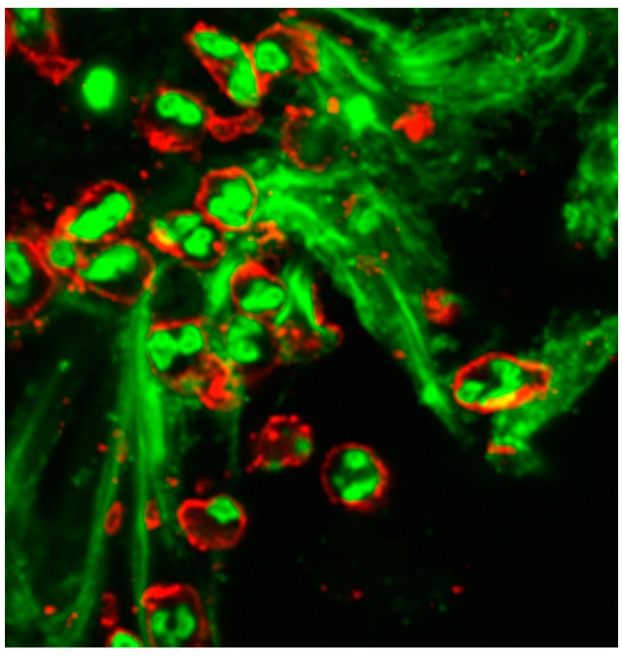
After stimulation with *S. epidermidis* biofilms, neutrophils also release DNA (stained in green), as shown by laser scan microscopy.

The general idea is that bacteria trapped within the DNA strands are more prone to be killed by neutrophil elastase, cathepsin G, and other bactericidal enzymes contained in NETs [75]. Recently, we were able to demonstrate that EPS and GroEL induce DNA release from neutrophils (Figure 4) [76]. It is unclear whether this release of DNA is an advantage for the host defences, because it has been described in the literature that extracellular DNA actually promotes and strengthens bacterial biofilm formation [55,77,78,79]. The complex interplay between staphylococcal biofilms and NETs also emerges from the finding that molecules involved in biofilm dispersal, such as nuclease [80], turn out to be capable of mediating the degradation of NETs [81]. Moreover, it should be pointed out that, because biofilms represent an abundant mass of living bacteria, the bacterial metabolic activity would be expected to affect the pH and oxygen levels of the milieu, reflecting on the immune response and NETs weaving [81].

Therefore, the biological consequence of DNA release in this context is still a matter of debate.

## 5. Biomaterials, Biofilms and Neutrophils

Finally, considering the role and significance of biomaterials in the biofilm-associated infections and the neutrophil-biofilm interaction, we highlight that biomaterials are *per se* prone to bacterial adhesion and favor biofilm formation [9]. Bacterial adhesion and biofilm production proceed in two steps. First bacterial adhesins mediate the interaction between the bacterium and the extracellular matrix proteins filming the biomaterial surface. Then, adhesin-mediated anchorage allows bacteria to cling to the biomaterial surface and to produce a biofilm. Physical and chemical characteristics of the biomaterial affect bacterial adhesion and biofilm formation. In this context, the development of anti-infective biomaterials and anti-adhesive surfaces have been regarded for a long time as the main strategy to prevent biofilm formation, perfecting more and more their anti-biofilm properties together with their safety for eukaryotic cells [9,82,83,84,85,86,87]. An interesting approach consists in modifying the surface of materials, which already possess the required bulk properties, making them refractory to bacterial adhesion and to biofilm formation [9,87]. Intrinsically bioactive materials or doped/blended/coated biomaterials with antibacterial substances are being progressively developed and optimized, as the new generation of *gently* *anti-infective biomaterials*, *i.e.*, materials that, besides being safe for eukaryotic cells and anti-infective towards bacteria, are also endowed with anti-inflammatory potential or other beneficial biological properties [88,89]. For the orthopaedic implants, the anti-infective materials should be designed to be able to oppose bacterial colonization and, at the same time, to support tissue repair [9]. Nanotechnologies and nanomaterials are opening new horizons [9,86].

Hänsch *et al.*, studied the behavior of polymorphonuclear neutrophils towards staphylococcal biofilms colonizing orthopaedic implant materials and showed that neutrophils are capable of penetrating biofilms and destroying them, although damaging the host tissues at the site of infection [30,90]. Neutrophils attempt to attack even the copious biofilms that grow onto biomaterials [91].

## 6. Conclusions

In conclusion, it can be reasonably supposed that the neutrophil defence against biofilm infections be effective in the majority of cases. Contrary to the current scientific trend, biofilms are not inherently protected from the host defences.

Neutrophils recognize, attack, and phagocytose biofilms, regardless of “opsonization”, which is required for phagocytosis of free-swimming bacteria. Activating molecules within the biofilm matrix, such as the bacterial heat shock protein GroEL, are recognized by neutrophils which are, thus, driven to a number of bactericidal strategies.

However, in the case of chronic biofilm-associated infections, the immune system is not capable of clearing biofilm and eradicating infection in a timely manner, which results in a persistent inflammatory response that leads to tissue damage. It follows that implant biofilm-associated infection tends to become chronic and the damage to endure. In the specific field of an orthopaedic implant-infection, this chronic inflammatory state stimulates a multiplicity of “unwanted side effects”, as osteoclast generation, bone degradation and, finally, implant-loosening [92]. However, hope comes from anti-infective biomaterials.
